# Elevation affects the ecological stoichiometry of Qinghai spruce in the Qilian Mountains of northwest China

**DOI:** 10.3389/fpls.2022.917755

**Published:** 2022-09-14

**Authors:** Huijun Qin, Liang Jiao, Yi Zhou, Jingjing Wu, Xichen Che

**Affiliations:** ^1^College of Geography and Environment Science, Northwest Normal University, Lanzhou, China; ^2^Key Laboratory of Resource Environment and Sustainable Development of Oasis, Gansu Province, Lanzhou, China

**Keywords:** stoichiometry, carbon, nitrogen, phosphorus, Qinghai spruce, soil

## Abstract

Environmental heterogeneity in temperature, moisture, and soil fertility caused by elevation gradients can affect the trade-offs in the survival strategies of tree species. There is uncertainty about the allocation of resources to different tissues of trees in response to the elevation gradient with respect to carbon (C), nitrogen (N), and phosphorus (P). Here, the C, N, and P content of leaves, branches, trunks, and thick and fine roots of *Picea crassifolia* (Qinghai spruce) and their stoichiometric changes across three different elevations were investigated in the Qilian Mountains. We found that N:P of Qinghai spruce was <14 in all tissues at most elevations, indicating that Qinghai spruce was more susceptible to N limitation. Meanwhile, the N content and N:P of Qinghai spruce each were significantly negatively correlated with temperature (*p* < 0.05), and its P content was lower at high elevation. The contribution of soil–climate interactions on the elevation gradient to each tissue type was 34.02% (leaves), 16.84% (branches), 67.78% (trunks), 34.74% (thick roots), and 49.84% (fine roots), indicating that interacting climate and soil factors on the elevation gradient predominately drove the C, N, and P content and stoichiometry variation in each tissue type of Qinghai spruce trees. The results of this study clarify that the elevation gradient regulates the elemental content and resource allocation in Qinghai spruce, providing basic data and an important timely reference for future forest management in the regions where coniferous trees grows. These findings also help improve our understanding of elevational patterns of forest ecosystem stoichiometry in arid and semiarid regions.

## Introduction

Ecological stoichiometry is the study of elemental mass balances, mainly carbon (C), nitrogen (N), and phosphorus (P) (Rong et al., 2015). In plants, C, N, and P are the primary nutrients that regulate important ecological functions, structures, and processes (Elser et al., 2010; Sardans et al., 2012; Zhang et al., 2018c). Plant stoichiometry is garnering more attention and is considered flexible both within and among species according to macro-scale studies, but this depends on factors of their growing environment, such as climate and soil resources (Sistla et al., 2015; Sun et al., 2017; Gong et al., 2020). Exploring the patterns of change in tree species C, N, and P stoichiometry is beneficial for understanding the allocation of resources in forest ecosystems and how they respond to environmental changes (Zhang et al., 2018c).

Mountain ecosystems have vital ecological functions due to their unique environmental and geological characteristics, serving as natural laboratories for studying ecological patterns along elevation gradients. In mountainous forest ecosystems, elevation as a key topographic factor strongly influences both climate and soil (Hoch and Körner, 2012; Macek et al., 2012; Peng et al., 2020) and plays a pivotal role in regulating C, N, and P ecological stoichiometry characteristics of ecosystem vegetation and soil (Fisher et al., 2013; Cui et al., 2021). In particular, elevation is the dominant factor determining habitat differences in mountainous areas, and the study of different elevations facilitates the formation of ecological theories. Several theories, ideas, and hypotheses have been proposed to characterize plant ecological stoichiometry, including those applied to elevational effects upon ecological stoichiometry, such as the temperature–biogeochemistry hypothesis (Reich and Oleksyn, 2004) and the temperature–plant physiology hypothesis (Reich and Oleksyn, 2004). The former argues that temperature affects the activity of soil microorganisms and thus the rates of soil N and P mineralization. Hence, the soil N and P contents are less available under low-temperature conditions, resulting in low leaf N and P contents in plants (Aerts and Chapin III, 1999; Reich and Oleksyn, 2004). The latter implies physiological processes in plants would be inhibited under low-temperature conditions, yet plants may initiate corresponding temperature-sensitive regulatory mechanisms to increase their N and P contents (Weih and Karlsson, 2001; Reich and Oleksyn, 2004). The aforementioned two hypotheses were proposed for leaves and have been tested accordingly in different studies. For example, the leaf N content of plants decreases with rising elevation in the Peruvian Amazon–Andes and northwestern Himalayas (Weg et al., 2009; Macek et al., 2012), supporting the temperature–biogeochemistry hypothesis. Numerous studies at regional, national, and even global scales have shown that the N and P contents of leaves increase with increasing latitude, mainly due to changes in temperature and precipitation along the latitudinal gradient, which is consistent with the temperature–plant physiology hypothesis (McGroddy et al., 2004; Han et al., 2005; Zhang et al., 2018a). It is not clear whether these two hypotheses are applicable to other tissue types of plants. Therefore, it is necessary to investigate other locally dominant or widespread species, as well as other plant parts, to further strengthen the study of the spatial distribution patterns of plant stoichiometry in order to verify which hypothesis better explains the changes in plant stoichiometric characteristics.

The ways in which stoichiometry is differentially partitioned among various tissues of plant species arise from a combination of their environmental elements and life history development (Zhao et al., 2016). These characteristics are not only governed by the effectiveness of soil fertility but also inextricably linked to the ecophysiological traits of plants. The roles of tissues in maintaining plant growth and development are not uniform, resulting in different resource requirements and distributions of C, N, and P among types of tissue (Yu et al., 2015). At the same time, the elemental and stoichiometric characteristics of tissues are closely related to their own growth and development, functional roles, and resource utilization strategies of the plant body, as well as its ability to adapt and respond to their local environment (Wright et al., 2004; Kerkhoff et al., 2006). Therefore, studying the stoichiometric characteristics of plant tissues is needed to better understand the mechanism of resource uptake and distribution in plants and for revealing the mechanisms by which species adapt to their environment. Stoichiometry homeostasis better reflects the physiological and biochemical adaptations of organisms to environmental changes (Hessen et al., 2004; Elser et al., 2010). Because distinct tissues of plants perform diverse functions, distinct tissues of the same species can also differ in their homeostasis characteristics. It has been reported that nutrient concentrations in leaves are restricted to provide optimal physiological traits in comparison to other non-photosynthetic tissues, meaning that leaves are generally more homeostatic (Zhang et al., 2018a; Wang et al., 2019d). Studies of tree seedlings and shrub plants have also found higher stoichiometric homeostasis in their leaves than roots (Garrish et al., 2010; Minden and Kleyer, 2014; Schreeg et al., 2014). Given that C, N, and P play different roles in plants, some resulting differences are expected in the content of different elements and their homeostasis characteristics. It has been shown that homeostasis is higher for N than for P in all plant species studied to date, indicating plants have a stronger ability to regulate the chemical element N, which is more abundant within their tissues (Yu et al., 2011; Li et al., 2016). By studying plant ecological stoichiometry homeostasis, insights can be gained into the adaptation strategies and ecological adaptations of plants to their habitats.

The Qilian Mountains are located in the northeastern part of the Tibetan Plateau, where they constitute an ecological barrier in northwest China. The Qilian Mountains feature significant differences in climate along their elevation gradient due to the unique climate and complex topography. Current studies on ecological stoichiometry in the Qilian Mountains have mostly focused on leaves and soils (Chen et al., 2016; Wang et al., 2019b; Cao et al., 2020; Yang et al., 2020; Kang et al., 2021; Niu et al., 2021); hence, there is a relative lack of studies investigating correlations between the stoichiometry of different plant tissues. More broadly, this has also largely limited our knowledge of other plant tissue stoichiometry and its application in ecological models (Chave et al., 2009). A comprehensive study on the ecological stoichiometry of various tissues of *Picea crassifolia* (Qinghai spruce) in the Qilian Mountains is thus imperative. Qinghai spruce is the dominant coniferous tree in the forest ecosystem of the Qilian Mountains, having a wide distribution and presumably pronounced elevation-dependent characteristics.

In this study, Qinghai spruce growing at different elevations was selected to analyze the distribution of the C, N, and P content and corresponding stoichiometry characteristics in its leaves, branches, trunk, and thick and fine roots. We addressed two fundamental questions: (1) What are the elevational patterns of stoichiometry for Qinghai spruce and does the tree stoichiometry differ across tissue types? (2) What are the main drivers of the C, N, and P content of Qinghai spruce tissues and their stoichiometry therein? We hypothesized the following: (1) Both the N and the P content may decline with rising elevation based on the temperature–plant physiology hypothesis; (2) the tissue stoichiometry characteristics of Qinghai spruce vary with elevation, but tissue homeostasis varies depending on different tissue functions and positions; and (3) the interaction between climate and soil along the elevational gradient has a greater impact on Qinghai spruce stoichiometry than climate and soil on the elevation gradient independently because both climate and soil can differentially influence the stoichiometry of plants (Zhang et al., 2018c; Joswig et al., 2022).

## Materials and methods

### Study area

**Figure 1:**
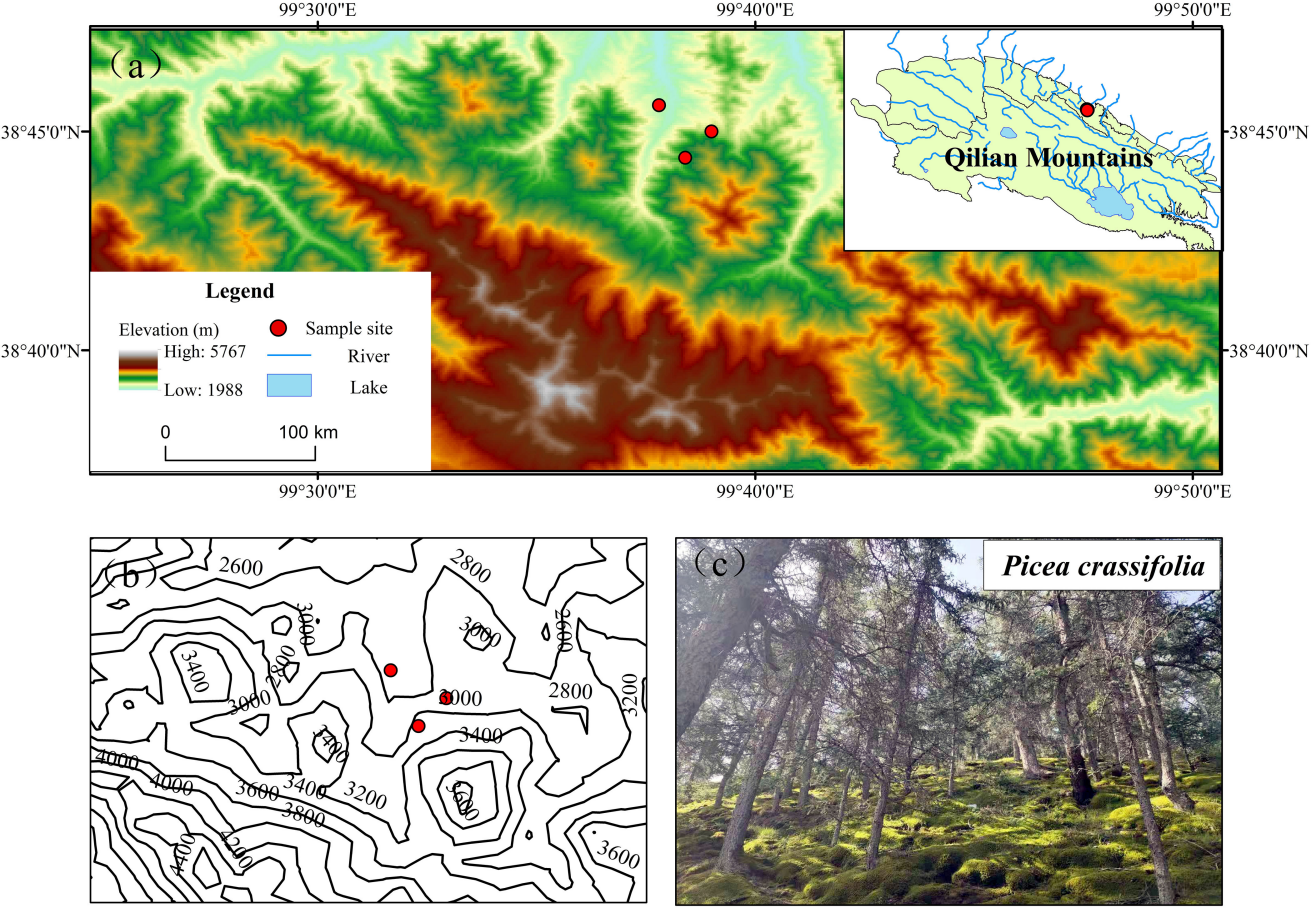
Locations of the three sampling sites forming the elevation gradient. Elevation maps of sampling sites **(a)**; contour maps **(b)**; photos of Qinghai spruce **(c)**.

The Qilian Mountains, located in the northeastern part of the Qinghai–Tibet Plateau, are an important mountain range in northwestern China. The three sampling sites (Table 1) at the Longchang River Nature Protection Station in the Qilian Mountains National Nature Reserve were selected as typical areas for the distribution of Qinghai spruce, where an alpine mountain forest–steppe climate prevails with obvious vertical gradients and horizontal differences in temperature and precipitation (Figure 1). With increasing elevation (from 2,600 to 3,200 m), the average annual temperature decreases from 1.14 to −2.16°C, while the average annual precipitation increases from 318.70 mm to 391.73 mm. Soil types in the watershed mainly include mountain chestnut calcium soil, mountain gray-brown soil, subalpine scrub meadow soil, and alpine cold desert soil. The Qinghai spruce is a major dominant tree species of the Qilian Mountains, the mainstay of water-conserving forests, found distributed on shady slopes at an elevation of 2,400–3,300 m. The dominant shrubs include *Potentilla fruticosa, Caragana jubata*, and *Salix gilashanica*, while the dominant herbs mainly include *Polygonum viviparum, Carex atrata*, and *Stipa krylovii*.

**Table 1:** Basic information of the sampling sites.

**Site**	**Elevation (m)**	**Latitude (°)**	**Longitude (°)**	**Mean annual temperature (°C)**	**Mean annual precipitation (mm)**
Low elevation	2,600	38.76	99.63	1.14	318.70
Middle elevation	2,900	38.75	99.65	−0.51	355.40
High elevation	3,200	38.74	99.64	−2.16	391.73

### Sampling and analysis

At each low (2,600 m), middle (2,900 m), and high-elevation (3,200 m) site, a 10-m × 10-m sampling plot was randomly set up in mid-July 2021 (Table 1). To minimize the effects of differences in light and needle surface temperature on the elements in the trees, sampling was always carried out at noon (Li et al., 2008). To minimize the bias of human disturbances, the sites were placed far away from human habitats. Our sampling plots were on the same slope aspect (north slope) with a little difference in slope, and the study area had a single tree species, only Qinghai spruce. There is no competition between tree species. A total of five Qinghai spruce plants that were upright, healthy, undamaged, non-isolated, and the same size (age, tree height, and diameter at breast height) were selected at each sampling site. The five trees we selected at each elevation are representative of the trees at that elevation. From each tree, leaf, branch, trunk, thick root (diameter > 5 mm), and fine root (diameter <2 mm) samples were collected. Leaf and branch samples were taken from unshaded mature branches that were ca. 2 years old, in the uphill direction. Tree cores were drilled at 1.3 m above ground (breast height) using growth cones for trunk sampling. The litter layer was removed from the topsoil, and samples of soil from 0 to 10 cm depth were collected at 2 m from each target tree. Soil was collected in four directions of each target tree, and these samples were mixed to yield a composite soil sample per tree (Liu and Wang, 2021). All plant samples were oven-dried at 105°C for 30 min on the day of collection, then dried to a constant weight at 65°C, ground through a 0.15-mm sieve using a hybrid ball mill (MM400, Retsch, Germany), and finally sealed for cryopreservation. Organic carbon in the Qinghai spruce tissue types and soil was determined using potassium dichromate sulfuric acid with the external heating method applied (Yeomans and Bremner, 1988). The N and P content was each determined using a fully automated chemical analyzer (Smartchem 200, Advanced Monolithic Systems, Graz, Italy) after heating to digest the samples.

### Data analysis

In the Qilian Mountains, the mean annual temperature and precipitation were calculated as follows (Zhao et al., 2005, 2006):


(1) $$\begin{array}{r} \text{MAT}=20.957-0.00549H-0.166\text{X}+0.0089\text{Y},{\text{R}}^{2}=0.98 \end{array}$$

(2) $$\begin{array}{r} \text{MAP}=1690.6235+0.119\text{H}-75.264\text{X}+12.405\text{Y},{\text{R}}^{2}=0.92 \end{array}$$

where *MAT* is the mean annual temperature, *MAP* is the mean annual precipitation, *H* is the elevation, *X* is the latitude, *Y* is the longitude, and *R*^2^ is the regression coefficient.

The proportion corresponding to the given tissue stoichiometry in Qinghai spruce was simply the stoichiometry of that tissue type divided by the summed stoichiometry of all tissue types.

The homeostasis index was calculated using the internal stability model: *y* = *c x*^1/*H*^ (Sterner and Elser, 2002), where *y* represents the concentration or ratio of chemical elements in the tissue of Qinghai spruce, *x* denotes the concentration or ratio of elements in soil, *c* is a constant, and *H* denotes the homeostasis index. The degree of homeostasis in plant tissues was classified as strictly homeostatic (*1/H* < 0), homeostatic (0 < *1/H* ≤ 0.25), weakly homeostatic (0.25 < *1/H* ≤ 0.5), weakly sensitive (0.5 < *1/H* ≤ 0.75), and sensitive (*1/H* > 0.75) (Makino et al., 2003; Persson et al., 2010).

Statistical analysis of the empirical data was implemented in SPSS 22.0 software (SPSS Inc., Chicago, IL, United States). After testing for normality, all non-normally distributed data were ln-transformed to improve their normality. All parameters in this study satisfied the assumption of homogeneity. One-way ANOVA and the least significant difference (LSD) test were used to compare significant differences in Qinghai spruce and soil stoichiometry at different elevations (three levels). Linear relationships between the stoichiometry of Qinghai spruce and environmental factors were assessed using Pearson's *r* correlation coefficient. The main drivers of inter-elevation Qinghai spruce stoichiometry were analyzed by CANOCO 5.0 by applying the redundancy analysis (RDA). The variance partitioning analysis (VPA) was conducted using the *varpart* function, in the “vegan” package in R v4.1.2, to quantify the contribution of climate and soil factors to the stoichiometry of various tissues of Qinghai spruce along the elevational gradient. Graphs were drawn by Origin 2022 software.

## Results

### Changes in soil stoichiometry at different elevations

**Figure 2:**
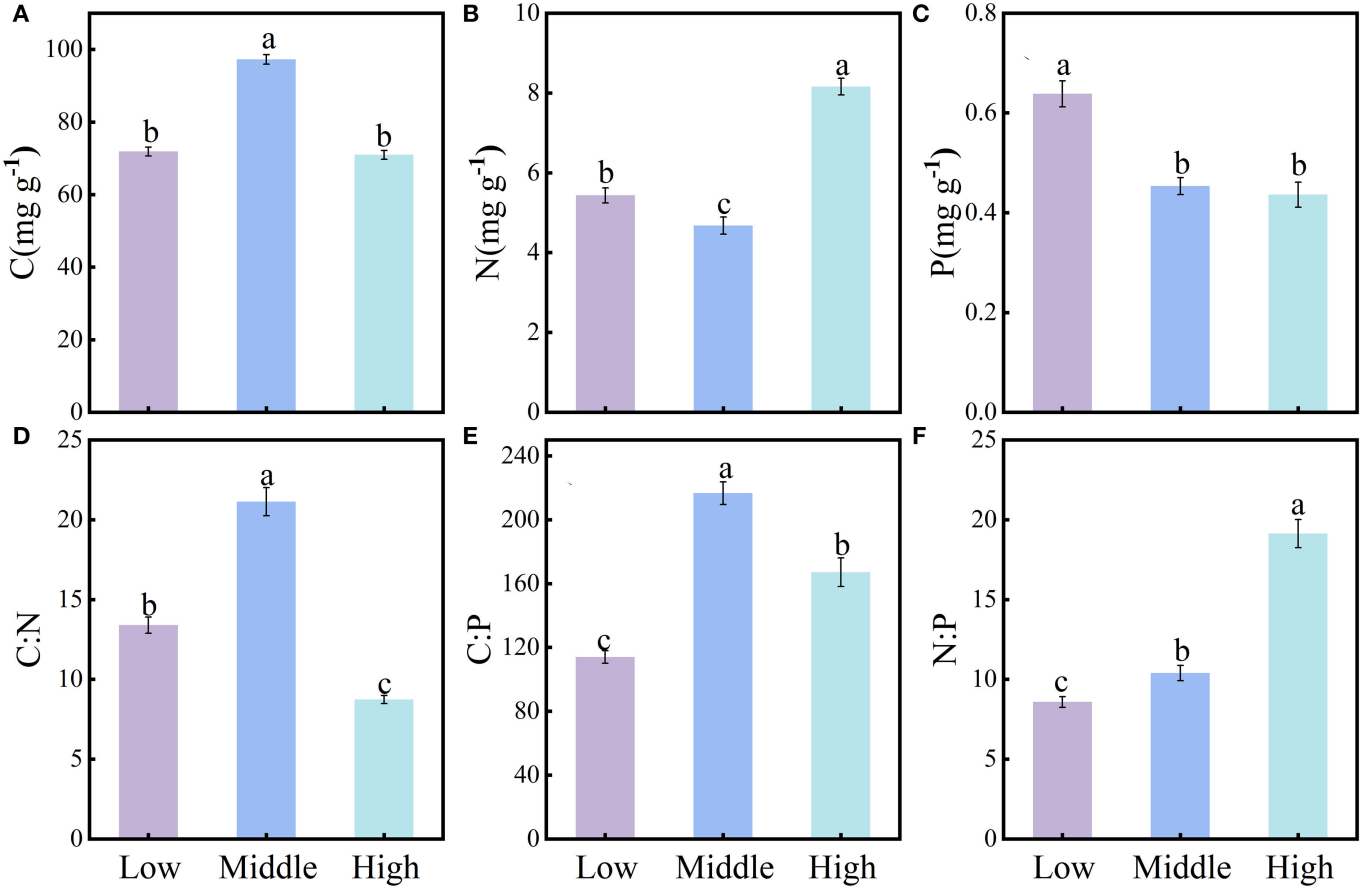
Characteristics of soil stoichiometry at different elevation levels (mean ± SE, different lowercase letters indicate significant differences among them (*P* < 0.05). Soil C, N, P content **(A–C)**; soil C:N, C:P, N:P **(D–F)**.

The content of soil C, N, and P and their stoichiometry differed significantly among the three elevations (Figures 2A–F). The soil C content, C:N, and C:P were significantly greater at middle elevation (C 97.23 ± 1.32 mg·g^−1^, C:N 21.13 ± 0.88 mg·g^−1^, C:P 216.64 ± 7.10 mg·g^−1^) than either low elevation (C 71.86 ± 1.23 mg·g^−1^, C:N 13.39 ± 0.50 mg·g^−1^, C:P 113.99 ± 3.95 mg·g^−1^) or high elevation (C 70.94 ± 1.23 mg·g^−1^, C:N 8.74 ± 0.25 mg·g^−1^, C:P 167.08 ± 8.96 mg·g^−1^) (Figures 2A,D,E). The soil N content was significantly lower at middle elevation and was ranked as follows: high elevation (8.16 ± 0.21 mg·g^−1^) > low elevation (5.43 ± 0.19 mg·g^−1^) > middle elevation (4.68 ± 0.21 mg·g^−1^) (Figure 2B). The soil P content decreased with increasing elevation along the gradient, being ranked as follows: low elevation (0.64 ± 0.03 mg·g^−1^) > middle elevation (0.45 ± 0.02 mg·g^−1^) > high elevation (0.44 ± 0.03 mg·g^−1^) (Figure 2C). Yet, N:P increased with elevation as follows: low elevation (8.58 ± 0.34 mg·g^−1^) > middle elevation (10.39 ± 0.48 mg·g^−1^) > high elevation (19.13 ± 0.89 mg·g^−1^) (Figure 2F).

### Distribution pattern of Qinghai spruce resources at different elevations

**Figure 3:**
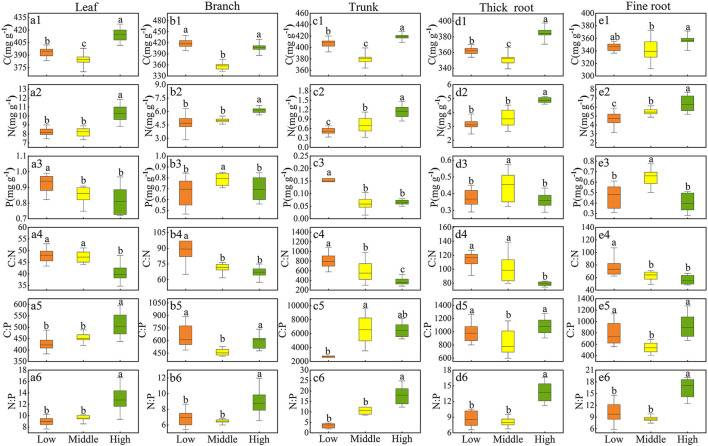
Range for the stoichiometry of Qinghai spruce across three elevation levels (low, middle, and high). Box plots show the 10–90th percentiles with median lines; different letters indicate significant differences among elevation levels at *P* < 0.05 (Tukey's test). C **(a1–e1)**, N **(a2–e2)**, P **(a3–e3)**, C:N **(a4–e4)**, C:P **(a5–e5)**, N:P **(a6–e6)** of each tissue.

The contents of C, N, and P and their stoichiometry in Qinghai spruce varied greatly among the three elevations (Figures 3a1–e6). The C content in all tissues was significantly lower at the middle elevation (*p* < 0.05) (Figures 3a1–e1). Evidently, at a high elevation, there was a greater N content than that at low and middle elevations (*p* < 0.05) (Figures 3a2–e2). Furthermore, leaves and trunks at high elevation contained less P than at low elevation (*p* < 0.05) (Figures 3a3,c3), while the P content of branches, thick roots, and fine roots at middle elevation significantly exceeded that at other elevations (*p* < 0.05) (Figures 3b3,d3,e3). C:N in all tissues decreased significantly with increasing elevation (*p* < 0.05) (Figures 3a4–e4); C:P in branches, and thick and fine roots was significantly lower at middle elevation, but C:P in leaves increased with increasing elevation as follows: low elevation (426.61 ± 8.91 mg·g^−1^) > middle elevation (452.77 ± 7.09 mg·g^−1^) > high elevation (510.04 ± 16.54 mg·g^−1^) (Figures 3b5,d5,e5). Likewise, C:P in the trunk increased with increasing elevation, showing that middle elevation (8492.39 ± 2358.81 mg·g^−1^) > high elevation (6506.89 ± 330.98 mg·g^−1^) > low elevation (2699.09 ± 438.14 mg·g^−1^) (Figure 3c5). N:P in all five tissue types was similar between low and middle elevations (*p* > 0.05), but N:P at high elevation significantly surpassed that at low and middle elevations (*p* < 0.05) (Figures 3a6–e6).

**Figure 4:**
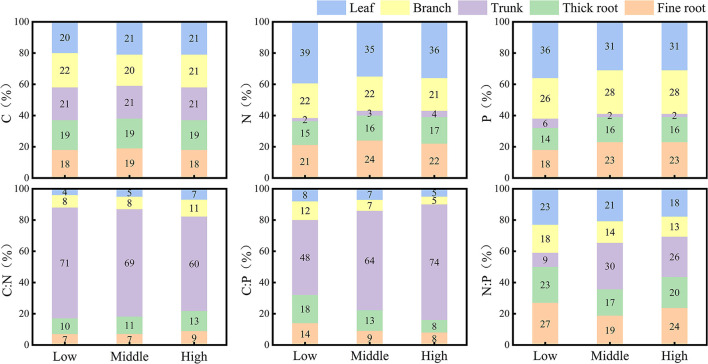
Distribution of stoichiometry measures among the tissue types of Qinghai spruce at different elevations.

At each elevation, the distribution of C in the tissues of Qinghai spruce did not vary much, falling within 18% and 22% (Figure 4). The distribution of N and P in tissues of Qinghai spruce at all three elevations attained their largest proportions in leaves (respectively, low elevation: 39 and 36%, middle elevation: 35 and 31%, high elevation: 36 and 31%) and their smallest proportions in trunk (respectively, low elevation: 2 and 6%, middle elevation: 3 and 2%, high elevation: 4 and 2%). The distribution of C:N and C:P in tissues of Qinghai spruce at all elevations always reached its largest proportion in the trunk (respectively, low elevation: 71 and 48%, middle elevation: 69 and 64%, high elevation: 60 and 74%). The distribution of N:P in Qinghai spruce at low elevation revealed the following patterns: fine roots (27%) > thick roots (23%) = leaves (23%) > branches (18%) > trunk (9%); at middle elevation, trunk (30%) > leaves (21%) > fine roots (19%) > thick roots (17%) > branches (14%); at high elevation, trunk (26%) > fine roots (24%) > thick roots (20%) > leaves (18%) > branches (13%).

### Relationships between the stoichiometry of Qinghai spruce and environmental factors

The stoichiometry of Qinghai spruce leaves, branches, trunks, and thick and fine roots were significantly correlated with the stoichiometry of soil, temperature, and precipitation (Supplementary Figure 1). Soil C was positively correlated with the P of fine roots and thick roots. The C content of leaves, trunk, and branches was each negatively correlated with soil C. For leaves, trunk, and thick roots, their content of C or N was positively correlated with soil N. The P content of leaves and trunk was positively correlated with temperature, but the N content and N:P of each tissue type were negatively correlated with temperature. Whereas precipitation was positively correlated with both the N content and N:P of each tissue type, it was negatively correlated with the P content of leaves and the trunk.

### Homeostasis of stoichiometry for Qinghai spruce

Qinghai spruce exhibited significant differences in its tissue stoichiometric homeostasis (Table 2). In terms of C content, all tissue types were categorized as strictly homeostatic, having 1/H_C_ values < 0. Concerning their N content, the leaves, branches, and fine roots were categorized as weakly homeostatic, having 1/H_N_ values of 0.39, 0.46, and 0.34, respectively; the trunk was weakly sensitive and thick roots categorized as sensitive. Regarding the P content and C:P, the branches, thick roots, and fine roots were categorized as strictly homeostatic; leaves were categorized as homeostatic, and the trunk was categorized as sensitive. The C:N homeostasis index of the trunk was 0.31, that is, weakly homeostatic, whereas it was homeostatic for other four tissue types. The N:P homeostasis indices of the leaf and branch, respectively, were 0.40 and 0.32, being weakly homeostatic; those of the trunk, thick roots, and fine roots belonged to the sensitive type, with values of 0.86, 0.55, and 0.63, respectively.

**Table 2:** Homeostasis of stoichiometry among five tissue types of Qinghai spruce.

**Parameters**	**1/H_C_**	**1/H_N_**	**1/H_P_**	**1/H_C:N_**	**1/H_C:P_**	**1/H_N:P_**
Leaf	−0.15	0.39	0.20	0.15	0.09	0.40
Branch	−0.43	0.46	−0.15	0.10	−0.37	0.32
Trunk	−0.23	1.20	1.69	0.31	1.83	0.86
Thick root	−0.18	0.61	−0.05	0.23	−0.21	0.55
Fine root	−0.07	0.34	−0.24	0.08	−0.42	0.63

1/H_C_ is the C homeostasis coefficient; 1/H_N_ is the N homeostasis coefficient; 1/H_P_ is the P homeostasis coefficient; 1/H_C:N_ is the C:N homeostasis coefficient; 1/H_C:P_ is the C:P homeostasis coefficient; and 1/H_N:P_ is the N:P homeostasis coefficient.

### Responses of Qinghai spruce to environmental factors

**Figure 5:**
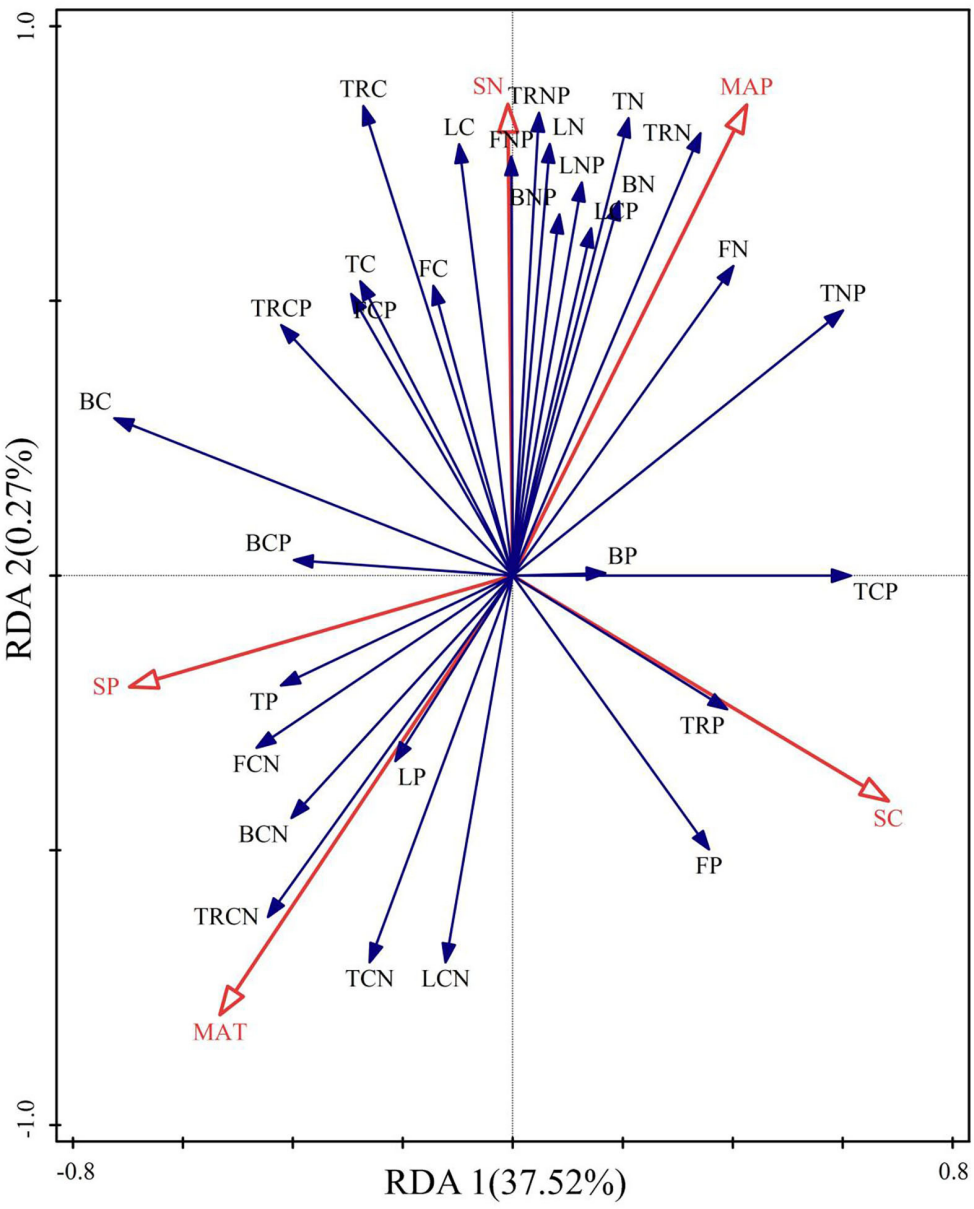
Redundancy analysis (RDA) of the relationships between the stoichiometry of Qinghai spruce and environmental factors. Red arrows represent environmental factors; tissue types are L, leaves; B, branches; T, trunk; TR, thick roots; F, fine roots. S denotes soil, MAT is mean annual temperature, and MAP is mean annual precipitation. CN represents C:N, CP denotes C:P, and NP represents N:P.

The constrained axes of the elevational RDA together explained 37.90% of the variation in Qinghai spruce stoichiometry, with the first and the second axis, respectively, accounting for 37.52 and 0.27% of the variation (Figure 5). Temperature and precipitation are the main drivers of resource allocation patterns in Qinghai spruce across elevations, showing that the stoichiometry of trees is strongly correlated with temperature and precipitation.

**Figure 6:**
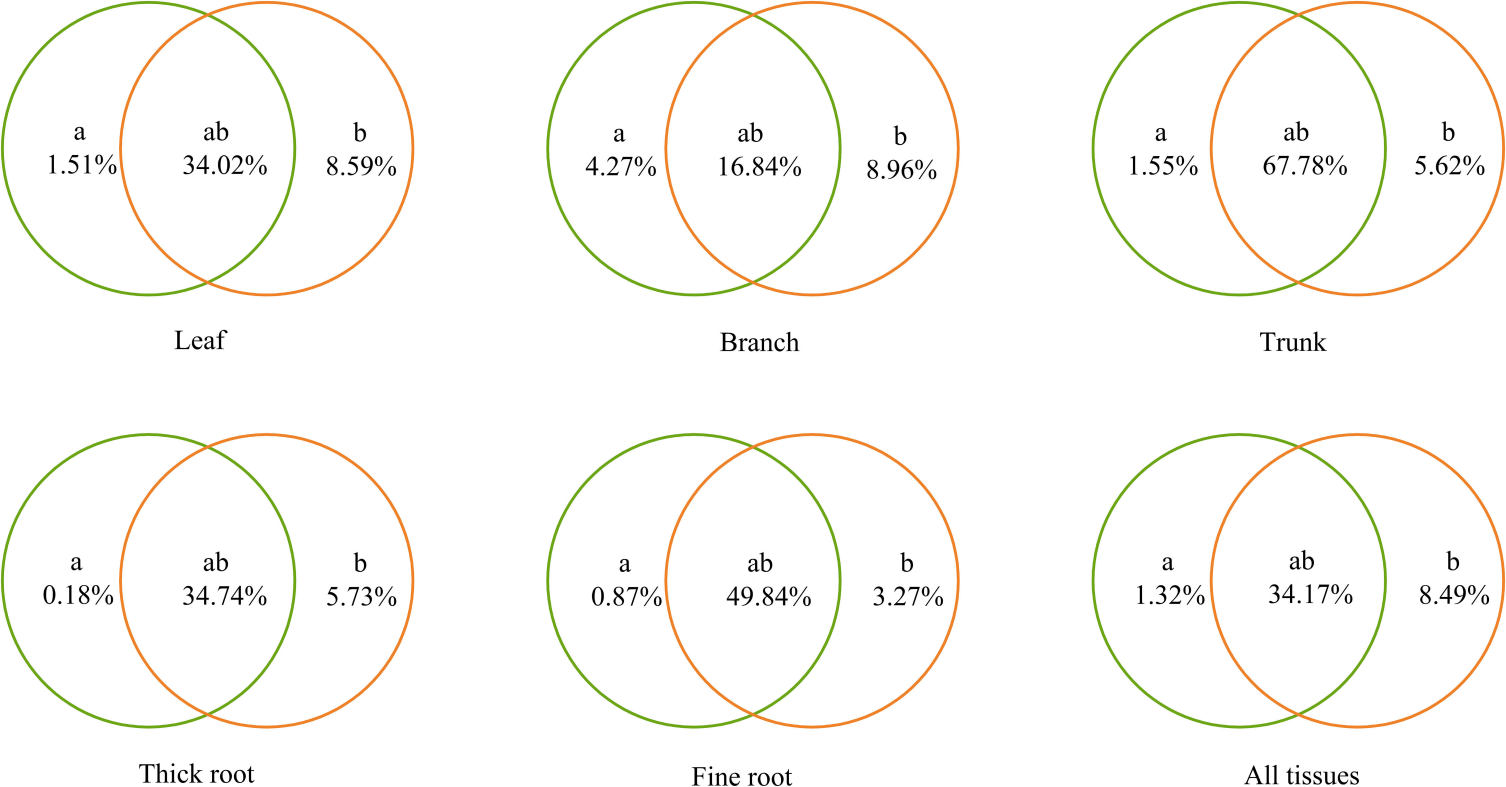
Variance partitioning analysis of the relationships between the stoichiometry of Qinghai spruce and environmental factors. “a” and “b” indicate the independent effects of soil and climate, respectively; accordingly, “ab” indicates the interactive effects of soil and climate.

Soil, climate, and their interaction together explained 30.07–74.95% of the variance in the stoichiometry characteristics of Qinghai spruce on the elevation gradient, according to the VPA (Figure 6). For all tissues of Qinghai spruce, their stoichiometry was affected most by soil and climate interactions on the elevation gradient (34.17%) > climate on the elevation gradient independently (8.49%) > soil on the elevation gradient independently (1.32%). Meanwhile, compared with belowground tissues, aboveground tissues of Qinghai spruce were affected more by soil and climate on the elevation gradient independently (leaves: 1.51 and 8.59%, branches: 4.27 and 8.96%, trunk: 1.55 and 5.62%, thick roots: 0.18 and 5.73%, fine roots: 0.87 and 3.27%).

## Discussion

### Elevational effects on the distribution of resources for Qinghai spruce trees

Environmental heterogeneity in temperature, moisture, and soil fertility caused by elevation gradients can affect tree growth. But due to the inherent variability of the regional climate and differences in tree physiology, much uncertainty is imbued in elevational effects on tree resource allocation (Sundqvist et al., 2013).

The C content of various tissues of Qinghai spruce first decreased and then increased with rising elevation (Figure 3). At low elevation, Qinghai spruce is often subjected to drought stress, which decreases its uptake rate of elements in the soil by the root system (Wang et al., 2017, 2019a). Qinghai spruce counteracts and adapts to that adversity by synthesizing the higher C-containing elements, that is, the C content of each tissue type increases in response to temperature increases. By contrast, the higher C content of Qinghai spruce at high altitude reflects its environmental adaptation strategy of increasing nonstructural carbohydrate content to resist low temperatures and reduce frost damage (Hoch et al., 2002; Millard et al., 2007; Hoch and Körner, 2012). The N content of Qinghai spruce tissues increased with increasing elevation, which is consistent with most studies (McGroddy et al., 2004; Han et al., 2005; Zhang et al., 2018b). On the one hand, this trend may be related to the increased N content in soil at greater elevation in this study area; there is more N available in soil at higher elevations for plants to uptake. On the other hand, it was consistent with the temperature–plant physiology hypothesis: that is, in order to compensate for the lower growth metabolic rate, the N content of each tissue was increased accordingly (Reich and Oleksyn, 2004). Unlike the trend for the N content, the P content of Qinghai spruce at high elevation was lower than that at the middle or low elevation, a trend consistent with some previous studies (Muller et al., 2017; Wang et al., 2018). This is probably because P in each tissue type is mainly derived from the uptake of soil P by roots, and in the study area, the soil P content declines significantly with rising elevation (Figure 2), such that the reduction of available plant P sources led to less P in each tissue. In addition, as the temperature decreases with increasing elevation, the activity of soil microorganisms is also affected, which in turn changes the mineralization rate of soil P. The lower availability of soil P can lead to an overall lower P content in all five tissues, which supports the temperature–biogeochemistry hypothesis (Reich and Oleksyn, 2004).

At the same time, the changed N and P content of Qinghai spruce along the elevation gradient resulted in greater N:P with elevation. This result is consistent with findings from a study of *Carex brevicuspis* in the Dongting Lake region of China (Li et al., 2018). The growth rate hypothesis states that high N:P often predicts a low growth rate of plants (Sterner and Elser, 2002). Accordingly, N:P increases with elevation suggests the growth rate of Qinghai spruce gradually slows down at higher elevations, in line with the temperature–plant physiology hypothesis (Reich and Oleksyn, 2004). Our research results deviate from the traditional stoichiometric theory that posits N and P elements respond consistently to environmental changes, indicating that plant N and P elements do not necessarily adhere to the stoichiometric theory in tandem, which reflects the unique adaptive mechanisms of Qinghai spruce to heterogeneous environments. In addition, there is a delicate balance between the composition of resources within the plant and the level of environmental resource availability. The dynamic changes in elemental stoichiometry ratios in plants can be used to determine the differences in the supply of resources in the environment and, thus, the types of limiting environmental elements for normal plant growth. It has been suggested that an N:P < 14 implies a N-limited condition, an N:P > 16 is considered a P-limited condition, and in the case 14 < N:P < 16, plants are both N and P co-limited. In our study, each tissue type of Qinghai spruce had an N:P < 14 at all three elevations, except for the trunk and fine roots with N:P > 16 at high elevation, which suggests that Qinghai spruce is more susceptible to N limitation.

### Resource allocation trade-offs among tissues in Qinghai spruce

The C, N, and P contents of different tissues of plants reflect the uptake and demand of resources and the ability of plants to adapt to different environments. The distribution of C among tissues for Qinghai spruce varies little along elevations (ranging from 18% to 22%) (Figure 4), and C was the most homeostatic among the three elements examined (Table 2). This is because C is the basic element constituting the largest proportion of the plant body. According to the theory of homeostasis, the elemental composition in organisms maintains a relatively stable state with the elemental supply of their surrounding environmental resources. This dynamic equilibrium is considered an essential characteristic of all life, such that the chemical composition of an organism should not change drastically with the changed chemical composition of its external environment. Hence, this fluctuation should be maintained within a certain narrow range, as seen for the C content of Qinghai spruce, it being the most stable across elevation and also between tissue types. The leaf is one of the most important functional tissues of plants, where both photosynthesis and the synthesis of many important compounds occur, such as chlorophyll, proteins, and nucleic acids. Hence, as C, N, and P are the most important elements in these biochemical processes, they attain their largest proportions in leaves.

A strong stoichiometric correlation between leaf and soil stoichiometry characteristics was found in our study, in line with previous studies (Lu et al., 2012; Zhang et al., 2018d). Moreover, the homeostasis of leaves was stronger than that of other tissues (Table 2), confirming that leaves should behave more adaptively under the influence of environmental change. This finding provides new evidence for our understanding of the importance of leaves in tree growth and reproduction dynamics (Yan et al., 2016; Wang et al., 2019c). As active organs, leaves can maintain the appropriate nutritional levels to ensure the optimal material and energy for plants. This mechanism is an important way by which trees adapt to external environmental changes and further meet the resource requirements for their growth and development.

C:N and C:P of the trunk surpassed those of other tissues. This disparity may be due to more N and P being allocated to active tissues, namely, leaves and branches, to meet the needs of plant growth and development processes (Sterner and Elser, 2002). The trunk is primarily responsible for structural support, so it contains more C than other tissue types. By contrast, leaves are responsible for photosynthesis and require sufficient N and P to synthesize various enzymes for biochemical reactions. Furthermore, branches are rich in N and P because they are responsible for the development of new tips and the exchange of resources. Nevertheless, for roots, their N and P contents exceed those of branches because roots connect and anchor trees to the soil matrix, and they are vital for absorbing water and mineral nutrients (Taiz and Zeiger, 2002). In general, the root system also needs enough N and P to support metabolism and to transfer the excess nutrients or products aboveground, to leaves and branches. Thus, the function of a tissue could determine the proportion of elements based on our results for resource allocation trade-offs among five tissue types in Qinghai spruce.

### Driving factors of resource allocation in Qinghai spruce

The growth and development of plants are closely related to their habitats, and changes in the stoichiometric characteristics of Qinghai spruce can reflect the nature of its response and adaptation to a heterogeneous environment. Along the elevation gradient, temperature and precipitation are undoubtedly key environmental factors affecting plant growth and development (Zheng et al., 2017). Moreover, previous studies found that the N or P content of plant tissues is mainly influenced by the spatiotemporal distributions of temperature and precipitation regimes (Yu et al., 2017). In our study, temperature and precipitation were also crucial drivers of spruce resource allocation in Qinghai, as inferred from the RDA (redundancy analysis) (Figure 5). Both the N content and N:P for each tissue type had significant negative correlations with temperature yet significant positive correlations with precipitation. On the contrary, for all tissue types, their C:N had significant positive and negative correlations with temperature and precipitation, respectively (Figure 5). These results indicate that temperature and precipitation play fundamental roles in regulating the resource allocation of Qinghai spruce trees (Liu et al., 2021). In the region of this study, dendrochronology research has shown that the radial growth of Qinghai spruce is highly sensitive to both temperature and precipitation (Tian et al., 2017; Gao et al., 2018). Accordingly, temperature and water effectiveness during the growing season should be paramount driving forces in maintaining the growth of Qinghai spruce trees and the sustainable development of the forest ecosystem in the Qilian Mountains.

The interconnectedness between plants and soil in their stoichiometry is an intrinsic regulatory mechanism for resource cycling in terrestrial ecosystems (Hobbie and Gough, 2010). The resource level of soil is also the main factor for determining the elemental concentration of plants. Accordingly, variation in its resources modulates the physiological characteristics of Qinghai spruce and, thus, the elemental content of its various tissues. The correlations performed here can reveal the coordination between the indicator variables of C, N, and P stoichiometric ratios of different tissues, which can better explain the coupling process between resources. For Qinghai spruce, there were mostly significant correlations between C, N, and P contents of each tissue vis-à-vis their soil stoichiometric ratios in the study area based on correlation and redundancy analyses (Figure 5; Supplementary Figure 1). We found that the N content and N:P across tissues were each significantly positively correlated with the soil N content (*p* < 0.05). The C, N, and P contents in leaves and trunks were significantly positively correlated with the soil C, N, and P content (*p* < 0.05). These results confirm the findings of previous studies. For example, N and P contents in plants were positively correlated with soil N and P contents in evergreen broadleaf forests of southwest China (Liu et al., 2010). In another work, both the P content and N:P of leaves were significantly correlated with soil P in the Dianchi Lake watershed (Yan et al., 2011). These results also indicate that the concentrations of soil resources are coupled with those of plant resources.

Climate and soil on the elevation gradient together influence the elevational patterns of Qinghai spruce stoichiometry (Figure 6). VPA showed the interaction of climate and soil on the elevation gradient contributed the most to each tissue type, followed by the independent effect of climate on the elevation gradient, with the independent effect of soil on the elevation gradient contributing the least to each tissue type stoichiometry. Compared with the independent effects of climate and soil, other studies have also found that climate and soil interactions contribute more to leaf stoichiometry and plant morphology in the Changbai Mountains of China (Zhao et al., 2014; Joswig et al., 2022). Moreover, climatic factors explained more of the variation than soil factors. Climatic changes along elevation gradients alter the hydrothermal conditions for the growth of Qinghai spruce, which might lead to differential resource allocation among different tissues. So, although soil provides the major nutrients (including trace elements) that sustain plant growth, it has less of impact on forest ecosystem functioning (Zhang et al., 2018c).

In this study, the C, N, and P contents in different tissues of Qinghai spruce on three elevation gradients and their response to elevation changes were investigated. Our sampling was typical, but considering the relative inadequacy of samples used at one specific site, future studies need to validate this finding in more settings and cover enough samples for better generalization. In addition, the fact that other currently unidentified factors, such as wind and root strategies of trees, affect tree stoichiometry patterns at different elevations should be considered in future studies. Our results help understand the role of the elevation gradient in regulating the elemental resources of Qinghai spruce and provide basic data and important references for future forest management in the region.

## Conclusion

Inter-elevation variation in the stoichiometry of tissues of Qinghai spruce trees in the Qilian Mountains was investigated. The results show that that tissue stoichiometry of this tree in addition to soil exhibited significant elevational spatial heterogeneity. However, leaf stoichiometry exhibited a strong homeostasis across different elevations, providing great potential for adaptation to future climate change. Also, Qinghai spruce is more susceptible to N limitation. It was also found that the C, N, and P contents and stoichiometry of Qinghai spruce tissues were influenced more by the interaction between climate and soil along the elevational gradient. Therefore, the conservation of Qinghai spruce should be enhanced to ensure the security and sustainability of forest ecosystems in the Qilian Mountains. In tandem, a reasonable amount of N fertilizer application is needed to improve the soil nutrient supply and safeguard nitrogen availability in forest management of the Qilian Mountains. Elevational effects on the ecological stoichiometry of Qinghai spruce in the Qilian Mountains could help us better understand the resource allocation patterns and ecological adaptation strategies conferring dominant plants of subalpine forest ecosystems in arid and semiarid regions.

## Data availability statement

The original contributions presented in the study are included in the article/Supplementary material, further inquiries can be directed to the corresponding author.

## Author contributions

HQ and LJ conceived and designed the study, wrote, reviewed, and edited the manuscript. HQ, YZ, JW, and XC collected the data. All authors have read and approved the manuscript.

## Funding

This research was funded by the National Natural Science Foundation of China (No. 41861006), CAS Light of West China Program (2020XBZG-XBQNXZ-A), the Research Ability Promotion Program for Young Teachers of Northwest Normal University (NWNU-LKQN2019-4), and 2021 Graduate Research Grant Program of Northwest Normal University (Grant/Award Number: 2021KYZZ02135).

## Conflict of interest

The authors declare that the research was conducted in the absence of any commercial or financial relationships that could be construed as a potential conflict of interest.

## Publisher's note

All claims expressed in this article are solely those of the authors and do not necessarily represent those of their affiliated organizations, or those of the publisher, the editors and the reviewers. Any product that may be evaluated in this article, or claim that may be made by its manufacturer, is not guaranteed or endorsed by the publisher.
