# Supplementary material for: Elevation affects the ecological stoichiometry of Qinghai spruce in the Qilian Mountains of northwest China
Source: Front Plant Sci. 2022 Sep 14;13:917755. doi: 10.3389/fpls.2022.917755 (PMC9515584; doi:10.3389/fpls.2022.917755)
Supplement: Supplementary file 1 [file Image_1.pdf]

Supplementary Figures

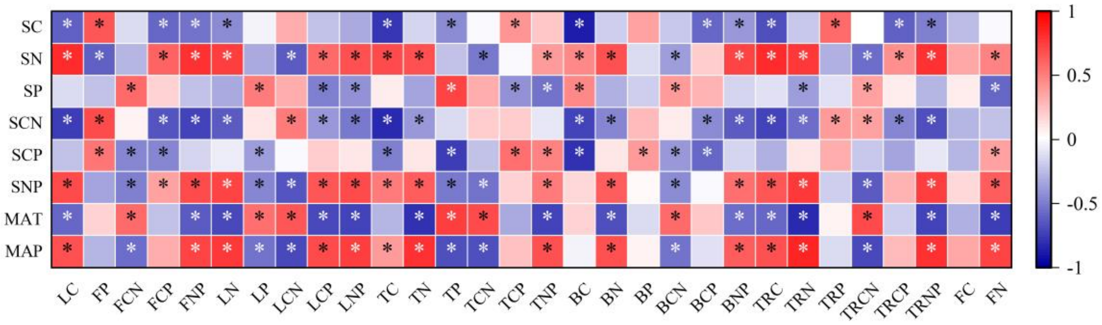

Supplementary Figure 1. Correlation between stoichiometry of Qinghai spruce and environmental factors (L represents leaves, B represents branches, T represents trunk, TR represents thick roots, F represents fine roots, S represents soil, MAT represents mean annual temperature, and MAP represents mean annual precipitation, CN represents the C:N ratio, CP represents the C:P ratio, and NP represents the N:P ratio).
